# Involvement of Class II Phosphoinositide 3-Kinase α-Isoform in Antigen-Induced Degranulation in RBL-2H3 Cells

**DOI:** 10.1371/journal.pone.0111698

**Published:** 2014-10-30

**Authors:** Kiyomi Nigorikawa, Kaoru Hazeki, Ying Guo, Osamu Hazeki

**Affiliations:** Graduate School of Biomedical & Health Sciences, Hiroshima University, Hiroshima, Japan; UPR 3212 CNRS -Université de Strasbourg, France

## Abstract

In this study, we present findings that suggest that PI3K-C2α, a member of the class II phosphoinositide 3-kinase (PI3K) subfamily, regulates the process of FcεRI-triggered degranulation. RBL-2H3 cells were transfected with shRNA targeting PI3K-C2α. The knockdown impaired the FcεRI-induced release of a lysosome enzyme, β-hexosaminidase, without affecting the intracellular Ca^2+^ mobilization. The release of mRFP-tagged neuropeptide-Y, a reporter for the regulated exocytosis, was also decreased in the PI3K-C2α-deficient cells. The release was increased significantly by the expression of the siRNA-resistant version of PI3K-C2α. In wild-type cells, FcεRI stimulation induced the formation of large vesicles, which were associated with CD63, a marker protein of secretory granules. On the vesicles, the existence of PI3K-C2α and PtdIns(3,4)P_2_ was observed. These results indicated that PI3K-C2α and its product PtdIns(3,4)P_2_ may play roles in the secretory process.

## Introduction

Mast cell activation mediated by the high-affinity receptor for IgE (FcεRI) is a key event in allergic inflammatory responses [1]. Cross-linking IgE-bound FcεRI triggers a rapid release of granule contents, including histamine, serotonin, and proteases. Previous studies have demonstrated the role of phosphoinositide 3-kinase (PI3K) in this process. The members of the PI3K family are lipid kinases that catalyze the phosphorylation of the 3-position of the inositol ring of phosphoinositides. PI3K can be grouped into three major classes (I, II, and III) based on their primary sequences, mechanism of regulation, and substrate specificities (for a review, please see Ref. [2]). In the process of FcεRI-mediated degranulation, the roles of class I subtypes, namely PI3Kδ and PI3Kγ, have been demonstrated [3], [4].

Although less investigated than class I subtypes, recent studies have shown that the class II subtypes of PI3K are involved in a variety of cell functions [5], [6]. Mammals possess three class II isoforms: PI3K-C2α, PI3K-C2β and PI3K-C2γ. PI3K-C2α and C2β are widely expressed in mammalian tissues. Human PI3K-C2γ showed a more restricted localization in the liver, prostate, breast and salivary glands [6], [7]. A previous study has demonstrated that siRNA against the class II isoform PI3K-C2β decreases the FcεRI-mediated Ca^2+^ influx and degranulation of bone marrow-derived mast cells (BMMCs) [8].

Another class II isoform, PI3K-C2α, has been implicated in several vesicle trafficking pathways [9]–[14]. As expected from the clathrin-binding motif in its N-terminal region [9], it was demonstrated that PI3K-C2α regulates clathrin-dependent endocytosis [9], [13]. Several studies have suggested the involvement of PI3K-C2α in exocytosis pathways, including translocation of glucose transporter type 4 to the plasma membrane of muscle cells, catecholamine release from adrenal chromaffin cells and insulin secretion from pancreatic β-cells [10]–[12], [14], [15]. However, the role of PI3K-C2α in the process of mast cell degranulation has not been reported to date. In the present study, we present results demonstrating that PI3K-C2α is involved in the exocytosis pathway of mast cells.

## Results

**Figure 1:**
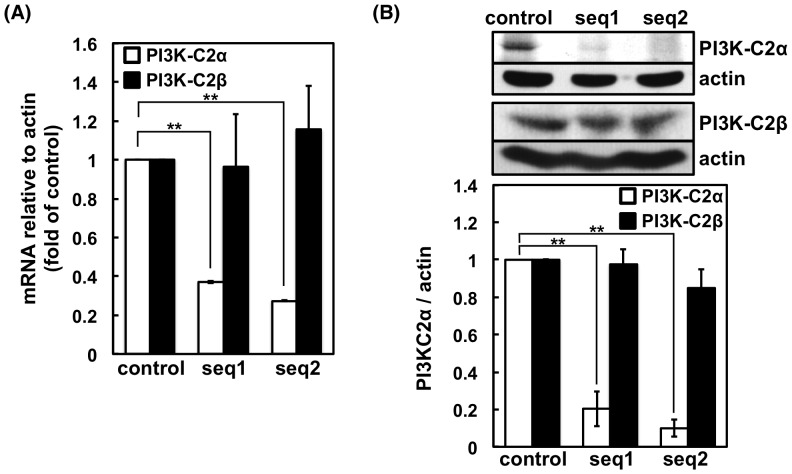
mRNA and protein expression levels of PI3K-C2α in control and shRNA-transfected RBL-2H3 cells. (A) Real-time PCR analysis of PI3K-C2α and PI3K-C2β in cells transfected with the control or shRNA expression vector. The mRNA levels are normalized against that of actin, and values relative to those of the control cells are shown as the means ± s.d. (n = 4). (B) Western blotting analysis of PI3K-C2α in cells transfected with control or shRNA expression vector. Representative blots from four separate experiments are shown. The protein expression levels are normalized against that of actin, and values relative to those of the control cells are shown as the means ± s.d. (n = 4).

We first examined the expression of PI3K-C2α and PI3K-C2β mRNA in RBL-2H3 cells. Reverse transcriptase-PCR with specific primers showed that PI3K-C2α and PI3K-C2β mRNA is expressed in RBL-2H3 cells (Figure S1). Because PI3K-C2β has been reported to regulate the FcεRI-induced Ca^2+^ influx and degranulation in BMMCs [8], we examined whether PI3K-C2α plays any role in the cells. To this end, we prepared RBL-2H3 cells expressing shRNA against PI3K-C2α. Two lines of cells that produce shRNA against the different sequences (seq1 or seq2) of PI3K-C2α were prepared. In the seq1- and seq2-targeted cells, the levels of PI3K-C2α mRNA were 37% and 27%, respectively, of the level observed in the control vector-transfected cells (Figure 1A). The PI3K-C2β mRNA was unaffected by the shRNA (Figure 1A). The protein levels of PI3K-C2α in the seq1- and seq2-targeted cells, as determined by western blotting with a specific antibody, were 20% and 9.9%, respectively, of the levels observed in the control cells (Figure 1B). The protein levels of PI3K-C2β were not significantly affected by the shRNAs (Figure 1B).

**Figure 2:**
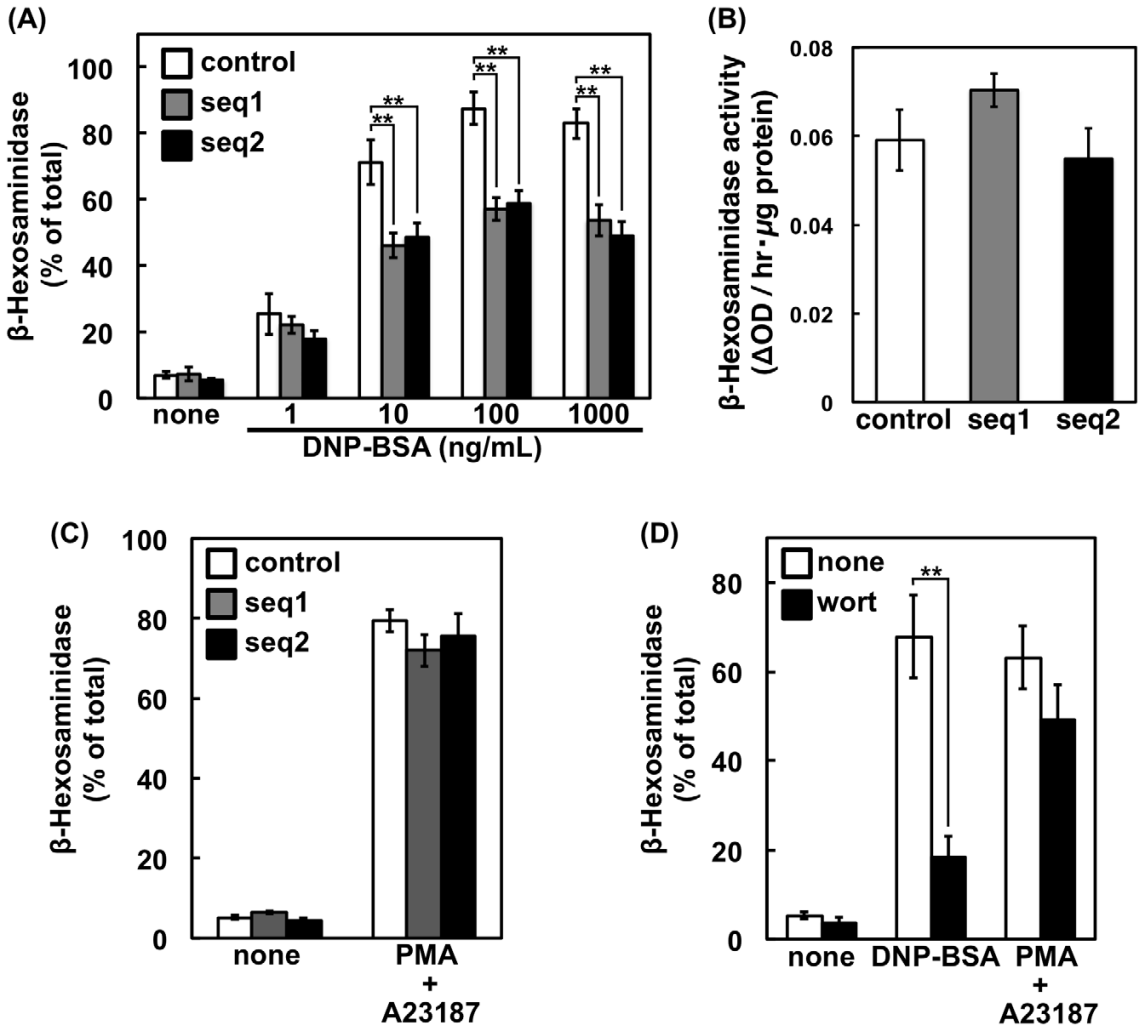
β-hexosaminidase release of PI3K-C2α-knockdown cells. (A) FcεRI-mediated β-hexosaminidase release. Control or PI3K-C2α-knockdown cells were sensitized with anti-DNP IgE and then stimulated with the indicated concentrations of DNP-BSA at 37°C for 10 min. After centrifugation, the β-hexosaminidase activity in the supernatant was determined and is shown as a percentage of the activity in the total cell lysate. The data are shown as the means ± s.d. (n = 4). (B) Content of β-hexosaminidase. The cells were solubilized for determination of β-hexosaminidase activity. The activities are expressed as the absorbance change at 405 nm per 1 µg of protein and are shown as the means ± s.d. (n = 4). (C) PMA/A23187-induced β-hexosaminidase release. The cells were pre-treated with 30 nM PMA at 37°C for 10 min and then stimulated with 1 µM A23187 for 10 min. The β-hexosaminidase activity was measured as in (A). The data are shown as the means ± s.d. (n = 4). (D) Effect of wortmannin. Sensitized or non-sensitized RBL-2H3 cells were incubated at 37°C with or without 30 nM PMA for 10 min. When added, 1 µM wortmannin was included during this period. The cells were then incubated with or without 100 ng/mL DNP-BSA or 1 µM A23187 for 10 min. The β-hexosaminidase activity was measured as in (A). The data are shown as the means ± s.d. (n = 3).

The effect of PI3K-C2α knockdown on the FcεRI-triggered release of a lysosomal enzyme, namely β-hexosaminidase, was examined (Figure 2A). The β-hexosaminidase release was decreased significantly in both PI3K-C2α-knockdown cells. The total granule content of β-hexosaminidase was unchanged by the shRNA transfection (Figure 2B). The results suggested that PI3K-C2α is required for efficient degranulation via FcεRI. When RBL-2H3 cells were treated with calcium ionophore and phorbol ester simultaneously, a significant amount of β-hexosaminidase was released. This response was, however, unaffected by PI3K-C2α knockdown (Figure 2C). The pan-PI3K inhibitor wortmannin, which inhibits PI3K-C2α with an IC_50_ value of 420 nM [16], efficiently decreased the FcεRI-triggered degranulation at 1 µM but did not alter the calcium ionophore/phorbol ester-induced response (Figure 2D).

**Figure 3:**
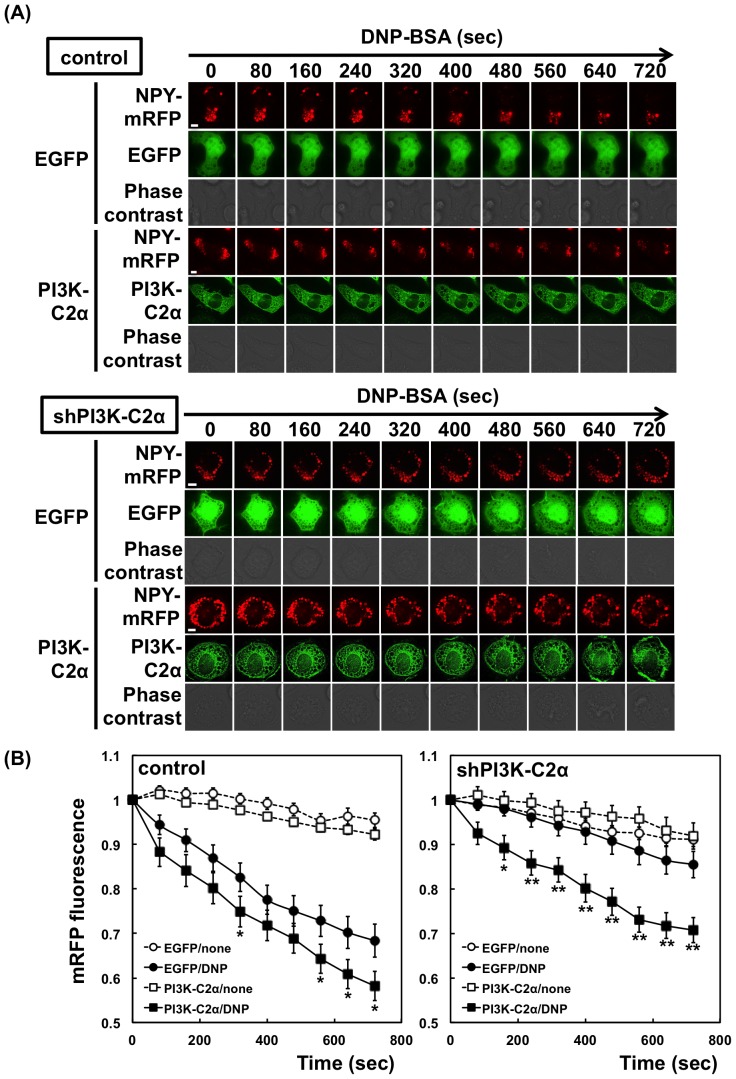
Neuropeptide Y release from PI3K-C2α-knockdown cells. (A) Effect of PI3K-C2α overexpression. The control or PI3K-C2α-knockdown (seq2) cells were transfected with NPY-mRFP. EGFP or shRNA-resistant EGFP-PI3K-C2α was transfected simultaneously. After 48 h, the cells were sensitized with anti-DNP IgE and then stimulated with 1 µM DNP-BSA. After stimulation, the red fluorescence in the cells showing the green fluorescence of EGFP was monitored. Scale bar = 5 µm. (B) Quantification of NPY-mRFP in cells. The intensities of mRFP fluorescence in control and PI3K-C2α-knockdown (seq2) cells were quantified and are shown relative to that at time zero. The data were obtained from six separate experiments (42 cells were examined in total) and are shown as the means ± s.e.m. *P<0.05, **P<0.01; the effect of PI3K-C2α expression is significant.

Neuropeptide Y (NPY) is a genuine reporter for the regulated exocytosis of mast cells [17]. In the experiments shown in Figure 3A, NPY-mRFP and EGFP- PI3K-C2α were transfected into RBL-2H3 cells. Upon stimulation, the mRFP fluorescence of the control cells decreased gradually, indicating the release of NPY from the cells. The NPY release from the PI3K-C2α-knockdown cells (shPI3K-C2α cells) was markedly slowed. The NPY release was then examined in the cells transfected with the shRNA-resistant PI3K-C2α construct. The overexpression of PI3K-C2α significantly increased the NPY-mRFP release from the control and shPI3K-C2α cells (Figure 3B), confirming the role of PI3K-C2α as a positive regulator of degranulation. A slight decrease in the mRFP fluorescence in the unstimulated cells (Figure 3B) may be due to the spontaneous release of NPY and fluorescence bleaching.

**Figure 4:**
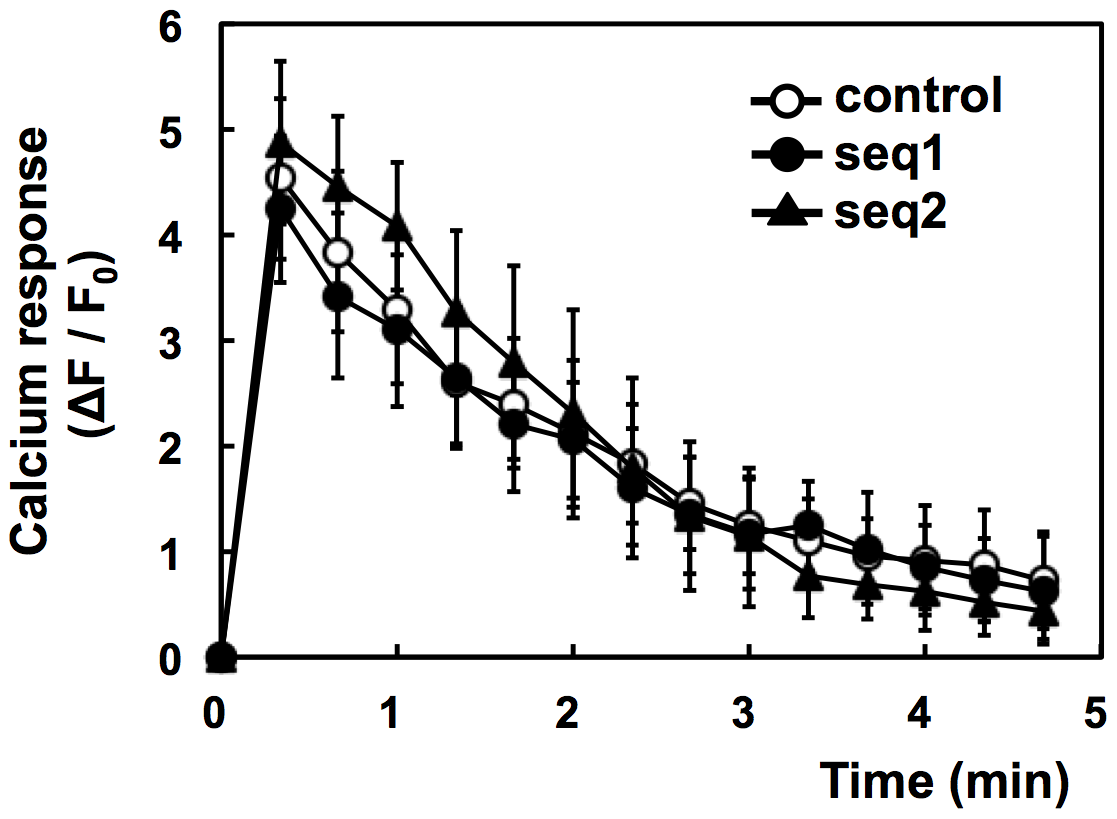
Failure of PI3K-C2α knockdown to inhibit antigen-induced calcium response. IgE-sensitized cells were incubated with Fluo-8 dye at 25°C for 20 min. The cells were washed and stimulated with 1 µM DNP-BSA at 30°C. The average fluorescence intensities (F) of the individual cells were monitored. The data are shown as ΔF/F_0_, where F_0_ is the basal F value obtained as the average intensity of the individual cells and ΔF is the difference between F and F_0_. The data were obtained from three separate experiments (24 cells were monitored in total) and are shown as the means ± s.d.

The increase in intracellular Ca^2+^ upon FcεRI stimulation is one of the key events triggering degranulation [18]. A recent study has shown that PI3K-C2β regulates the FcεRI-stimulated activation of the KCa3.1 channel and degranulation in BMMCs [8]. Thus, we investigated whether the down-regulation of PI3K-C2α influences the intracellular Ca^2+^ mobilization following FcεRI stimulation. Ca^2+^ imaging analysis revealed a rapid increase in intracellular Ca^2+^ upon FcεRI stimulation in the control and PI3K-C2α-knockdown cells, with no difference between these cell lines (Figure 4). The above results suggested that PI3K-C2α regulates the FcεRI-triggered degranulation by a mechanism different from that induced by PI3K-C2β.

**Figure 5:**
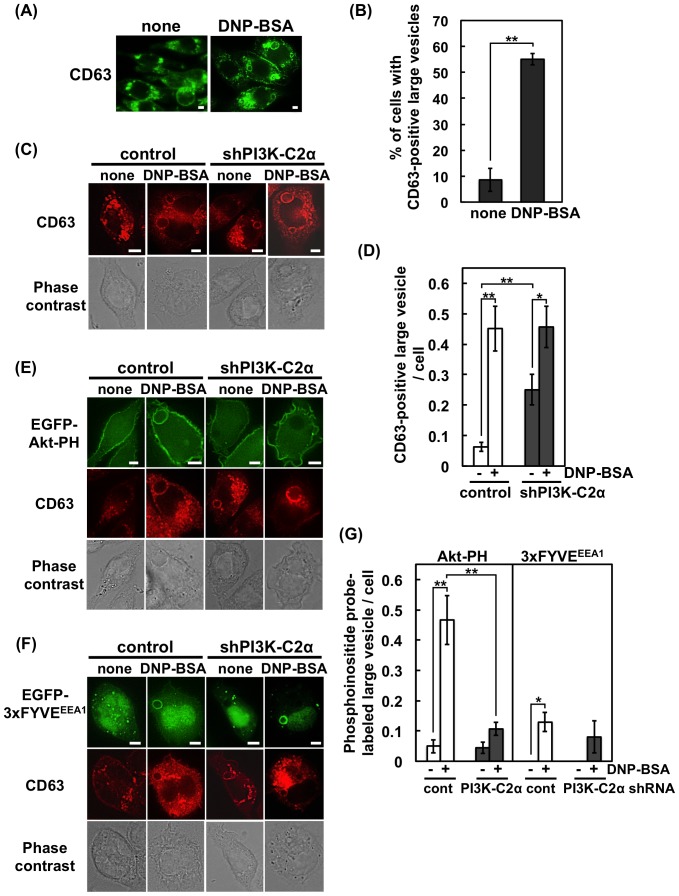
Existence of CD63 and PtdIns(3,4)P_2_ on large vesicles in FcεRI-stimulated RBL-2H3 cells. (A) Existence of CD63 on large vesicles. RBL-2H3 cells were stimulated, fixed and prepared for staining with anti-CD63 and Alexa 488-labeled anti-mouse IgG antibodies. Scale bar = 5 µm. (B) Numbers of cells containing CD63-positive large vesicles (>2.5-µm diameter). For each experimental condition, 75 cells were analyzed. The data are shown as the means ± s.e.m. from four separate experiments. (C) Existence of CD63 on large vesicles in control and PI3K-C2α-knockdown cells. Scale bar = 5 µm. (D) Numbers of CD63-positive large vesicles (>2.5-µm diameter) per cell. For each experimental condition, 161 cells were analyzed. The data are shown as the means ± s.e.m. from four separate experiments. (E) Absence of PtdIns(3,4)P_2_ in CD63-positive large vesicles in PI3K-C2α-knockdown cells. The cells were transfected with EGFP-Akt-PH. Before or after FcεRI stimulation, the cells were fixed. Scale bar = 5 µm. (F) Presence of PtdIns(3)P on CD63-positive large vesicles in control and PI3K-C2α-knockdown cells. The cells were transfected with EGFP-3×FYVE^EEA1^. Scale bar = 5 µm. (G) Numbers of large vesicles containing PtdIns(3,4)P_2_ or PtdIns(3)P. The cells were transfected with EGFP-Akt-PH or EGFP-3×FYVE^EEA1^. The number of vesicles displaying EGFP fluorescence was counted. For each experimental condition, 210 cells were analyzed. The data are shown as the means ± s.e.m. from three separate experiments.

We then examined the effect of PI3K-C2α knockdown on the granule dynamics following FcεRI stimulation. In the experiment shown in Figure 5A, RBL-2H3 cells were stimulated with antigen, fixed, and then stained with a specific antibody against CD63, a marker protein of secretory granules (also known as lysosome-associated membrane protein 3, LAMP3). In the resting state, most of the CD63-positive granules had a diameter smaller than 1 µm, as has been reported in a previous study [19]. Upon antigen stimulation, CD63-positive large vesicles (>2.5-µm diameter) appeared in more than a half of the cell population (Figure 5A, B) likely due to the granule-granule fusion and the granule swelling that occur during the process of exocytosis [20]–[23].

Previous studies have indicated that PI3K-C2α can catalyze the phosphorylation of PtdIns and PtdIns(4)P to generate PtdIns(3)P and PtdIns(3,4)P_2_, respectively [13]. To gain insight into the function of PI3K-C2α, the PH domain of Akt (Akt-PH) that recognizes PtdIns(3,4)P_2_ and PtdIns(3,4,5)P_3_ was transfected into RBL-2H3 cells. In the unstimulated cells, a large amount of EGFP-labeled Akt-PH was found on the plasma membrane (Figure 5E). This signal may be due to the products of class I PI3K because it can be observed similarly in the control and shPI3K-C2α cells. Upon the FcεRI stimulation, CD63-positive large vesicles associated with Akt-PH appeared in the control cells. Such vesicles were rarely observed in the shPI3K-C2α cells (Figure 5E, G). Similar results were obtained when the tandem PH domain of TAPP1 (2×TAPP1-PH) was used as a selective probe for PtdIns(3,4)P_2_ (Figure S2A, C). A similar experiment was then performed with the PtdIns(3)P probe 3×FYVE^EEA1^ (the FYVE domain of EEA1). The FcεRI stimulation tended to increase the 3×FYVE^EEA1^ association with the CD63-positive large vesicles, but this response was observed even in the absence of PI3K-C2α (Figure 5F, G). We next used another PtdIns(3)P probe, 2×FYVE^Hrs^ (the tandem FYVE domain of Hrs), which has been successfully used to monitor the PI3K-C2α-mediated PtdIns(3)P production at secretory vesicles in PC12 cells [14]. The experiment with 2×FYVE^Hrs^ produced similar results to that with 3×FYVE^EEA1^ (Figure S2B, C). The C215S mutant of the 2×FYVE^Hrs^, which does not bind PtdIns(3)P [24], did not associate with the vesicles (Figure S2D). These results suggested that PI3K-C2α and its product PtdIns(3,4)P_2_ may play some roles in the process of FcεRI-mediated degranulation.

**Figure 6:**
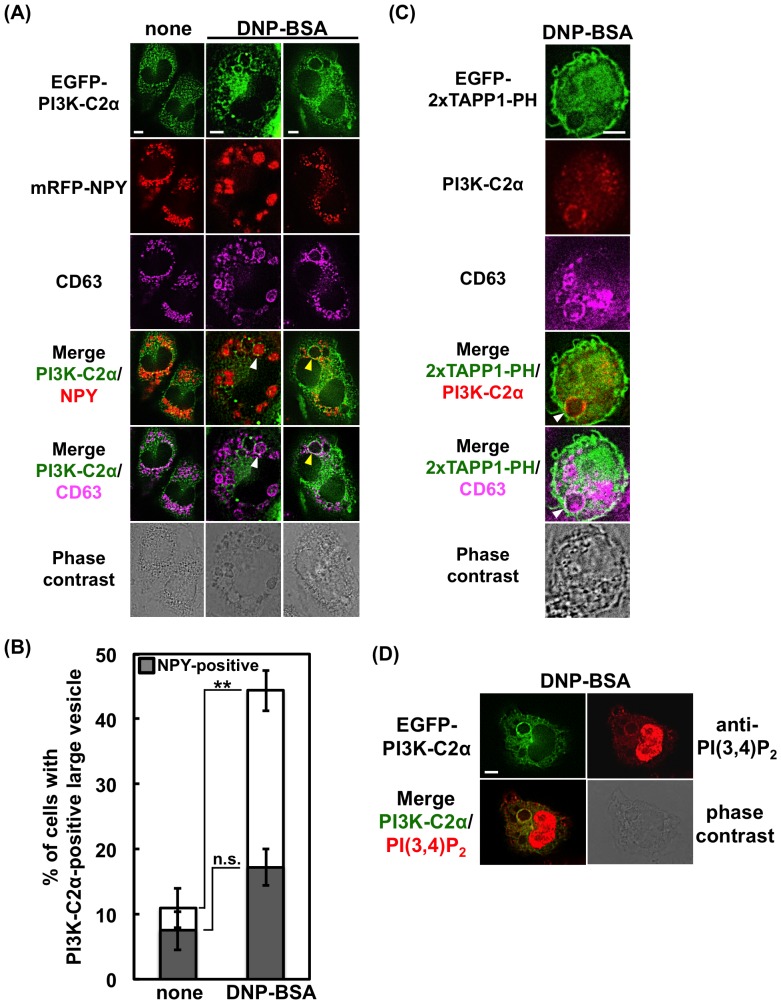
Existence of PI3K-C2α and PtdIns(3,4)P_2_ on large vesicles in FcεRI-stimulated RBL-2H3 cells. (A) Existence of PI3K-C2α on CD63-positive large vesicles containing or not containing NPY-mRFP. RBL-2H3 cells were transfected with EGFP-PI3K-C2α and NPY-mRFP. The cells were fixed before or after FcεRI stimulation, and stained with anti-CD63 and Alexa 647-labeled anti-mouse IgG antibodies. The arrowheads indicate the EGFP-PI3K-C2α and CD63-positive vesicles; the white indicates the NPY-mRFP-containing vesicle, while the yellow indicates the empty vesicle. Scale bar = 5 µm. (B) Numbers of cells containing EGFP-PI3K-C2α and CD63-positive large vesicles (>2.5-µm diameter). Solid bar indicates the number of cells possessing one or more vesicles containing NPY-mRFP. For each experimental condition, 106 cells were analyzed. The data are shown as the means ± s.e.m. from three separate experiments. (C) Existence of endogenous PI3K-C2α and PtdIns(3,4)P_2_ on CD63-positive large vesicles. RBL-2H3 cells were transfected with EGFP-2×TAPP1-PH. After stimulation, the cells were fixed, treated with anti-PI3K-C2α and anti-CD63, and then stained with Alexa 555-labeled anti-rabbit and Alexa 647-labeled anti-mouse IgG antibodies. The arrowhead indicates the EGFP-2×TAPP1-PH, PI3K-C2α and CD63-positive vesicle. Scale bar = 5 µm. (D) Existence of PI3K-C2α and PtdIns(3,4)P_2_ on large vesicles. RBL-2H3 cells were transfected with EGFP-PI3K-C2α. After stimulation, the cells were fixed and stained with anti-PtdIns(3,4)P_2_ and Alexa 647-labeled anti-mouse IgG antibodies. Scale bar = 5 µm.

We then examined the intracellular localization of PI3K-C2α. In the experiment shown in Figure 6A, RBL-2H3 cells were transfected with EGFP-tagged PI3K-C2α and mRFP-tagged NPY and stained with the specific CD63 antibody. Before stimulation, PI3K-C2α was found on the intracellular small vesicles and ruffling membrane. After antigen stimulation, PI3K-C2α-associated CD63-positive large vesicles appeared in about 45% of the cell population (Figure 6A, B). Nearly 40% of this cell population possessed one or more NPY-containing vesicles, whereas the others possessed empty vesicles only (Figure 6B). It has been reported that granules, which had released their contents, are recycled (60%) or collapse to the plasma membrane (40%) in RBL-2H3 cells [22]. Therefore, the above results suggest the association of PI3K-C2α with the secretory granules.

In the experiment shown in Figure 6C, RBL-2H3 cells were transfected with EGFP-2×TAPP1-PH, and the endogenous PI3K-C2α and CD63 were stained with specific antibodies. The result showed that PtdIns(3,4)P_2_ locates on the large vesicles that associate with PI3K-C2α and CD63. The existence of PtdIns(3,4)P_2_ on the large vesicles was confirmed when a specific antibody against PtdIns(3,4)P_2_ was used for the analysis (Figure 6D). These results suggested that PI3K-C2α and its product PtdIns(3,4)P_2_ play some roles in the process of FcεRI-mediated degranulation.

## Discussion

PI3K-C2α has been implicated in several exocytosis pathways [10]–[12], [14], [15]. In the present study, we found the first demonstration of the involvement of PI3K-C2α in the antigen-induced degranulation pathway. We observed that the expression of shRNAs against PI3K-C2α significantly reduced the FcεRI-mediated β-hexosaminidase release (Figure 2). The release of NPY-mRFP, a reporter of the regulated exocytosis of mast cells [17], was increased by PI3K-C2α overexpression and was impaired in the PI3K-C2α-deficient cells (Figure 3). The impaired exocytosis in PI3K-C2α-deficient cells was significantly rescued by shRNA-resistant PI3K-C2α expression (Figure 3).

A class II PI3K isoform, PI3K-C2β, has been reported to regulate the FcεRI-induced activation of the KCa3.1 channel in BMMCs [8]. In T cells, this isoform is required for the TCR-stimulated activation of the KCa3.1 channel and Ca^2+^ influx [25]. In the former, siRNA against PI3K-C2β impairs the FcεRI-induced increase in intracellular Ca^2+^ and degranulation [8]. In the present study, we observed that impaired degranulation in the PI3K-C2α knockdown cells did not accompany a decrease in the Ca^2+^ response (Figure 4). The result indicates that PI3K-C2α and PI3K-C2β share different roles in the FcεRI-regulated degranulation pathway. The biochemical background for this functional difference should be examined.

Secretory granules in mast cells have been shown to enlarge upon antigen-stimulation due to granule-granule fusion and/or granule swelling [20]–[23]. In the present study, we observed the formation of CD63-positive large vesicles in the FcεRI-stimulated RBL-2H3 cells (Figure 5A and B). CD63, also known as LAMP3, is a membrane protein containing a lysosome-targeting domain [26]. In RBL-2H3 cells, CD63 co-localizes with the SNARE proteins syntaxin3 and VAMP7, both of which are involved in the fusion of secretory granules with plasma membranes [27]. In BMMCs from the CD63-knockout mice, FcεRI-induced degranulation was impaired [28]. In the present study, we observed the existence of PI3K-C2α on the CD63-positive large vesicles (Figure 6A), suggesting a link between PI3K-C2α and CD63 during FcεRI-mediated degranulation. On this point, it is intriguing to note that the PMA/ionomycin-induced degranulation, which is unaffected in the shPI3K-C2α cells (Figure 2C), is not impaired in CD63-knockout mice [28]. It has been reported that mast cells have three types of secretory granule subset [29]. The subset driven by ionomycin and PMA is reported to be distinct from the FcεRI-driven one [30]. PI3K-C2α may be involved in the exocytosis of FcεRI-specific granules.

Previous studies have indicated that class II PI3Ks have the *in vitro* ability to phosphorylate PtdIns and PtdIns(4)P to generate PtdIns(3)P and PtdIns(3,4)P_2_, respectively [16]. It has been reported that PtdIns(3)P is the sole product in the presence of Ca^2+^ as the divalent cation [6], [31], [32]. In contrast, a recent study demonstrated that the immunopurified PI3K-C2α preferentially produces PtdIns(3,4)P_2_ in the presence of both Mg^2+^ and Ca^2+^ [13]. Regarding the *in vivo* product of class II PI3Ks, previous studies have yielded conflicting data on the preference of PtdIns(3)P or PtdIns(3,4)P_2_ as a product [10]–[14]. In the present study, we observed the association of Akt-PH and 2×TAPP1-PH, markers of PtdIns(3,4)P_2_, with CD63-positive large vesicles, and this association was impaired markedly in the shPI3K-C2α cells (Figure 5E, G, S2A and C). The presence of PtdIns(3,4)P_2_ on the vesicles of normal cells can be confirmed by blotting with a specific antibody (Figure 6D). The results suggest that PtdIns(3,4)P_2_ plays a functional role in PI3K-C2α-induced degranulation.

We observed that PtdIns(3)P-positive large vesicles were formed in the antigen-stimulated RBL-2H3 cells (Figure 5G, S2C). The number of these vesicles was lower than that of the PtdIns(3,4)P_2_-positive vesicles (<0.13±0.03 and 0.47±0.07 in each control cell, respectively). PI3K-C2α knockdown did not impair the formation of the PtdIns(3)P-positive vesicles (Figure 5G, S2C). It has been reported that crosslinking FcεRIs induces membrane ruffling [33], which in turn increases the formation of a macropinosome, which has a diameter of 0.5–10 µm and follows a fate similar to that of endosomes [34]. Early endosomes are reported to be rich in PtdIns(3)P. Thus, the PtdIns(3)P-positive vesicles observed in our present study are considered to be a macropinosome.

As described above, our results suggest that PI3K-C2α promotes antigen-induced degranulation through PtdIns(3,4)P_2_ production. This speculation is in accordance with previous studies that suggest that PI3K-C2α specifically phosphorylates PtdIns(4)P in the process of clathrin-mediated endocytosis [13] and in insulin-stimulated MIN6 cells [11]. In the former case, the role of PtdIns(3,4)P_2_ to recruit SNX9 to the clathrin-coated pits has been demonstrated [13]. In contrast, several studies including those on the insulin secretion of pancreatic β-cells [15], the catecholamine release from adrenal chromaffin cells [12], [14] and the glucose transporter type 4 translocation in insulin-stimulated cells [10] have indicated that PI3K-C2α regulates the later steps of exocytosis by producing PtdIns(3)P. A further study to identify the effector molecules of PtdIns(3,4)P_2_ in mast cells is warranted to confirm the role of PI3K-C2α in FcεRI-mediated degranulation.

## Materials and Methods

### Reagents and antibodies

Albumin-dinitrophenyl (DNP-BSA), anti-dinitrophenyl IgE, 4-nitrophenyl N-acetyl-β-D-glucosamide and A23187 were purchased from Sigma-Aldrich Japan (Tokyo, Japan). Wortmannin and PMA were obtained from Kyowa Medex (Tokyo, Japan) and Funakoshi (Tokyo, Japan), respectively. The monoclonal antibodies against PI3K-C2α, PI3K-C2β, CD63 (AD1) and PtdIns(3,4)P_2_ were obtained from BD Biosciences (San Jose, CA, USA), AbD Serotec (Kidlington, Oxford, UK) and Echelon Biosciences (Salt Lake City, UT, USA), respectively. The polyclonal antibody against PI3K-C2α was obtained from GeneTex (Irvine, CA, USA). Alexa Fluor 488-, 555- and 647-conjugated F (ab)_2_ fragments of goat anti-mouse and anti-rabbit IgG were purchased from Cell Signaling Technology (Danvers, MA, USA).

### Cell lines

Rat basophilic leukemia-2H3 (RBL-2H3) cell line [35] was purchased from ATCC (CRL-2256). RBL-2H3 cells were maintained in adherent cultures in RPMI 1640 medium (Nacalai tesque, Kyoto, Japan) supplemented with 10% FBS in a humidified atmosphere of 5% CO_2_ at 37°C.

### Cells lacking PI3K-C2α

Oligonucleotides with the sequence targeting PI3K-C2α were cloned into the pH 1 vector downstream of the H1 RNA promoter as previously described [36] to express short hairpin RNA (shRNA). The shRNA sequences used were as follows: seq1, 5′-GCACTGGAGAATGAAATAA-3′; seq2, 5′-GGTACATGATGACTTGAAT-3′. The insert sequences were confirmed by sequencing, and the plasmids were transfected into RBL-2H3 cells at 250 V/950 µF (Gene Pulser II; Bio-Rad, Hercules, CA, USA). At Seventy-two hours after transfection, puromycin (1 µg/ml) was added to the cells, and the incubation was continued to select for resistant cells. The control cells were prepared as described above using the pH 1 vector containing a 400-bp stuffer sequence instead of the target sequence.

### Plasmids

cDNAs encoding human PI3K-C2α (GenBank accession number NM_002645.2), human NPY (NM_000905.3) and FYVE domain of mouse Hrs (NM_001159328.1) were amplified by reverse transcriptase-PCR from ovary total RNA (Agilent Technologies, Santa Clara, CA, USA) or total RNA of Raw264.7 cell line (ATCC) using primers possessing additional nucleotide sequences convenient for subcloning. The cDNA constructs of PI3K-C2α and NPY were subcloned into the expression vectors pCAGGS-EGFP and pCAGGS-mRFP, respectively. For the rescue experiments, silent mutations were introduced into the target sequence of PI3K-C2α constructs (5′-GGTACATGATGAtcTGAAT-3′) using PCR mutagenesis. The cDNA of FYVE domain of mouse Hrs (residues 147–223) tandemly ligated to pCAGGS-EGFP with the linker QGQGS separating the two FYVE domains [37]. A point mutation (C215S) introduced into both FYVE domains using PCR mutagenesis and the following mutagenic primers: 5′-GCGCGTGTGTGAGCCCaGCTATGAGCAGCTGAAC-3′ and the complementary primer. The plasmids encoding EGFP, EGFP-Akt-PH, EGFP-3×FYVE^EEA1^ and EGFP-2×TAPP1-PH were kindly provided by Dr. Takehiko Sasaki (Akita University, Akita, Japan).

### RNA isolation and RT-PCR

The total RNA from RBL-2H3 cells was isolated with Sepasol-RNA I Super G (Nacalai tesque, Kyoto, Japan), and first-strand cDNA was synthesized by reverse transcription using random primers and M-MLV reverse transcriptase (Promega, Madison, WI, USA). The PCR analysis of the resulting cDNA was performed using a Flash SYBR Green qPCR kit (Thermo Scientific, Waltham, MA, USA) and the following forward and reverse primers: PI3K-C2α, 5′-AGGCTAGTGGATCAGCCAGA-3′ and 5′-GTTTGAGAGGAACCCGGCAT-3′; PI3K-C2β, 5′-TGAGCAAGATCTGGGTGCAG-3′ and 5′-TTACGCAGTGTCTCGGCATT-3′; actin, 5′-TTTGCAGCTCCTTCGTTGC-3′ and 5′-TCGTCATCCATGGCGAACT-3′. PCR was performed on a PikoReal Real-Time PCR System (Thermo Scientific). The data were normalized according to the expression of actin, and the relative mRNA expression levels were calculated.

### Degranulation assay

Degranulation was determined as the release of the granule marker β-hexosaminidase. RBL-2H3 cells cultured on a 96-well plate were sensitized with anti-DNP IgE (100 ng/ml) for 1 h in culture medium. IgE-sensitized RBL-2H3 cells were washed twice with PIPES buffer (119 mM NaCl, 5 mM KCl, 1 mM CaCl_2_, 0.4 mM MgCl_2_, 5.6 mM glucose, 1 mg/ml BSA, and 25 mM PIPES, pH 7.6) and then incubated at 37°C for 10 min. After addition of the indicated concentrations of DNP-BSA or vehicle, the reaction plate was maintained at 37°C for 10 min. For receptor-independent stimulation, unsensitized cells were incubated in PIPES buffer with PMA (30 nM) for 10 min and then stimulated with A23187 (1 µM) for 10 min. The cell pellets were solubilized in PIPES buffer containing 1% Triton X-100. The β-hexosaminidase activities of the supernatants and the solubilized pellets were measured by incubating with 4-nitrophenyl N-acetyl-beta-D-glucosaminide (Sigma) in sodium citrate (pH 4.5) for 1 h at 37°C. Then, 0.5 M Tris(hydroxymethyl)aminomethane was used to stop the reaction, and the absorbance was read at 405 nm. Degranulation is expressed as a percentage of β-hexosaminidase activity in the supernatant divided by the total (supernatant plus pellet) activity.

### Western blotting

RBL-2H3 cells were lysed in lysis buffer consisting of 25 mM Tris-HCl (pH 7.6), 100 mM NaCl, 1 mM EDTA, 1 mM sodium orthovanadate, 30 mM NaF, 200 µM PMSF, 20 µM p-APMSF, 2 µM leupeptin, 2 µM pepstain, and 1% Nonidet P-40. After centrifugation (15,000 rpm for 10 min), aliquots of the supernatant were mixed with SDS-PAGE sample buffer and boiled for 5 min. The peptides were separated by SDS-PAGE and transferred onto Immobilon-P membranes (Millipore, Bedford, MA, USA). After blocking, the membranes were incubated with the indicated antibody (1∶1,000 dilution), washed, and then incubated with horseradish peroxidase-conjugated secondary antibody (1∶10,000 dilution). The secondary antibody was detected using an enhanced chemiluminescence detection system (Perkin-Elmer, Waltham, MA, USA).

### Measurement of changes in intracellular Ca^2+^

IgE-sensitized RBL-2H3 cells in multi-well, glass-bottom dishes (Matsunami Glass) were incubated with Fluo-8 dye (AAT Bioquest Inc., Sunnyvale, CA, USA) in RPMI 1640 medium supplemented with 25 mM HEPES and 0.1% FBS at 25°C for 20 min. The cells were washed twice with PIPES buffer, stimulated with 1 µM DNP-BSA, and then observed under a fluorescence microscope (BZ-9000; Keyence, Tokyo, Japan) at 30°C using a GFP-BP filter. Fluorescence images were collected every 20 s, and the fluorescence intensities (F) of the individual cells were quantified with a BZ-II analyzer (Keyence). The data are shown as ΔF/F_0_, where F_0_ is the fluorescence intensity before stimulation and ΔF is the difference between F and F_0_. The data were obtained from three separate experiments (24 cells were monitored in total).

### Transfection

The transfection of plasmid DNA was conducted using the Neon transfection system (Life Technologies Co., Carlsbad, CA, USA) according to the manufacturer's instructions. In brief, RBL-2H3 cells (10^5^ cells) were washed with PBS and resuspended in 10 µL of R buffer containing 1 µg of plasmid DNA. The resuspended cells were then transferred into a gold tip and electroporated by two pulses at 1200 V for 20 ms followed by incubation in growth media without antibiotics for 48 h.

### Immunocytochemistry

IgE-sensitized RBL-2H3 cells in glass-bottom dishes were stimulated with 1 µg/mL DNP-BSA for 3 min. After washing with PBS, the cells were fixed with PBS containing 4% formaldehyde for 15 min. The cells were permeabilized with PBS containing 0.3% Triton X-100 and 0.5% BSA for 60 min, incubated with anti-CD63 (1∶500 dilution) or anti-PtdIns(3,4)P_2_ (1∶200 dilution) and/or anti-PI3K-C2α antibody (1∶250 dilution) at 4°C overnight and then incubated with Alexa 488- or Alexa 647-labeled (Fab′)_2_ fragment of goat anti-mouse IgG (1∶1,000 dilution) and/or Alexa 555-labeled (Fab′)_2_ fragment of goat anti-rabbit IgG (1∶1,000 dilution) for 2 h at room temperature. For the cells transfected with NPY-mRFP, the permeabilization was done with PBS containing 0.05% saponin and 3% BSA for 10 min. Microscopic analysis was performed using the Keyence BZ-9000 with CFI Plan Apo VC60xH lens (Keyence, Osaka, Japan). To obtain improved optical resolution along the z-axis, z-stacks were captured at 1-µm steps over a Z-axis distance of 3 µm, and individual planes as well as the sum projection of entire stacks were compared.

### Release of Neuropeptide-Y

RBL-2H3 cells expressing neuropeptide Y (NPY)-mRFP were transfected with either EGFP or shRNA-resistant EGFP-tagged PI3K-C2α. The cells in glass-bottom dishes were sensitized with IgE and then stimulated with 1 µg/mL DNP-BSA. The intensity of the red fluorescence in the cells showing EGFP expression was monitored under a fluorescence microscope using a Texas Red filter.

### Statistical analysis

Statistical significance was determined using unpaired, two-tail distribution, student's t-test. Data indicated with one asterisk or two asterisks have values of *P<0.05 or **P<0.01, respectively.

## Supporting Information

Figure S1 **mRNA expression of class II PI3K in RBL-2H3 cells.** PCR using RBL-2H3 cDNA as the template was performed with primers specific for PI3K-C2α or PI3K-C2β.
(TIF)

Figure S2 **Existence of CD63 and PtdIns(3,4)P_2_ on large vesicles in FcεRI-stimulated RBL-2H3 cells.** (A) Absence of PtdIns(3,4)P_2_ in CD63-positive large vesicles in PI3K-C2α-knockdown cells. The cells were transfected with EGFP-2×TAPP1-PH. Before or after FcεRI stimulation, the cells were fixed. Scale bar = 5 µm. (B) Presence of PtdIns(3)P on CD63-positive large vesicles in control and PI3K-C2α-knockdown cells. The cells were transfected with EGFP-2×FYVE^Hrs^. Scale bar = 5 µm. (C) Numbers of large vesicles containing PtdIns(3,4)P_2_ or PtdIns(3)P. The cells were transfected with EGFP-2×TAPP1-PH or EGFP-2×FYVE^Hrs^. The number of vesicles displaying EGFP fluorescence was counted. For each experimental condition, 150 cells were analyzed. The data are shown as the means ± s.e.m. from three separate experiments. (D) Specificity of 2×FYVE^Hrs^. RBL-2H3 cells were transfected with EGFP-2×FYVE^Hrs^ or its C215S mutant. The cells were fixed before or after FcεRI stimulation. Scale bar = 5 µm.
(TIF)
