# Supplementary figures and images for: Involvement of Class II Phosphoinositide 3-Kinase α-Isoform in Antigen-Induced Degranulation in RBL-2H3 Cells
Source: PLoS One. 2014 Oct 30;9(10):e111698. doi: 10.1371/journal.pone.0111698 (PMC4214793; doi:10.1371/journal.pone.0111698)

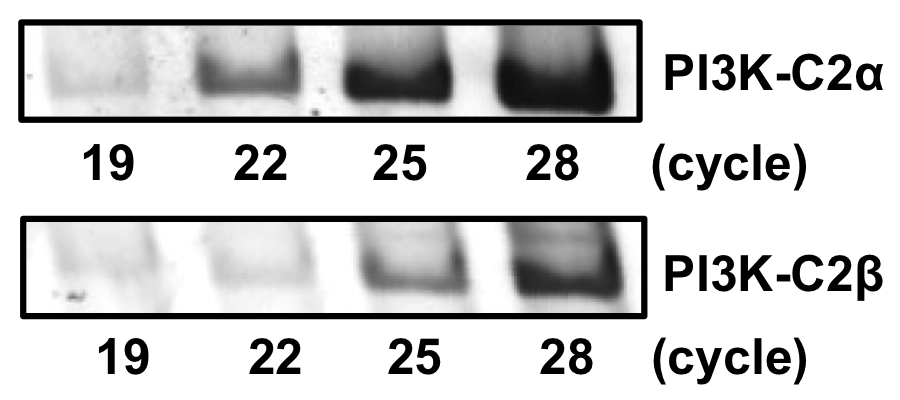

Supplement: Figure S1 — mRNA expression of class II PI3K in RBL-2H3 cells. PCR using RBL-2H3 cDNA as the template was performed with primers specific for PI3K-C2α or PI3K-C2β. (TIF) [file pone.0111698.s001.tif]

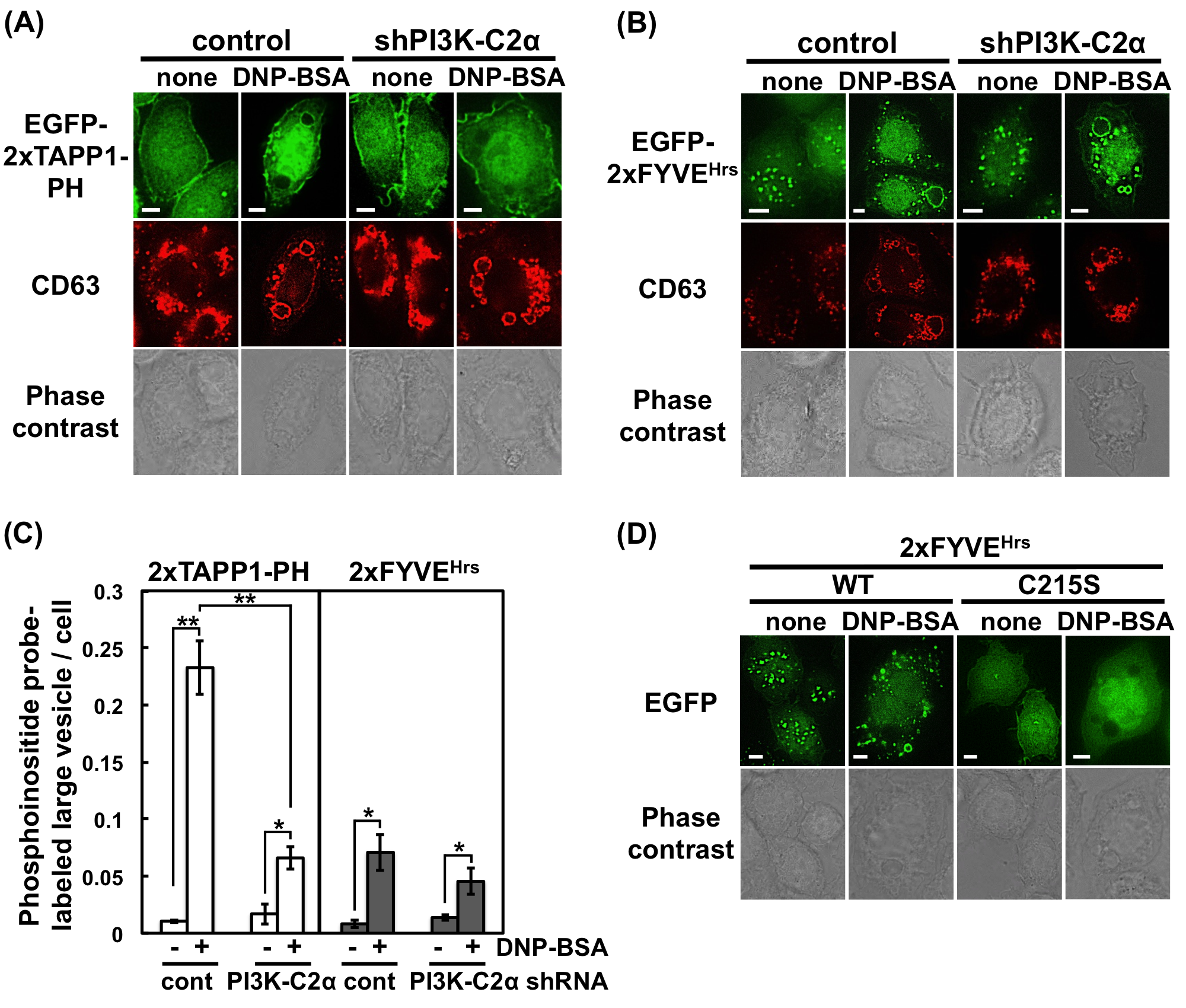

Supplement: Figure S2 — Existence of CD63 and PtdIns(3,4)P2 on large vesicles in FcεRI-stimulated RBL-2H3 cells. (A) Absence of PtdIns(3,4)P2 in CD63-positive large vesicles in PI3K-C2α-knockdown cells. The cells were transfected with EGFP-2×TAPP1-PH. Before or after FcεRI stimulation, the cells were fixed. Scale bar = 5 µm. (B) Presence of PtdIns(3)P on CD63-positive large vesicles in control and PI3K-C2α-knockdown cells. The cells were transfected with EGFP-2×FYVEHrs. Scale bar = 5 µm. (C) Numbers of large vesicles containing PtdIns(3,4)P2 or PtdIns(3)P. The cells were transfected with EGFP-2×TAPP1-PH or EGFP-2×FYVEHrs. The number of vesicles displaying EGFP fluorescence was counted. For each experimental condition, 150 cells were analyzed. The data are shown as the means ± s.e.m. from three separate experiments. (D) Specificity of 2×FYVEHrs. RBL-2H3 cells were transfected with EGFP-2×FYVEHrs or its C215S mutant. The cells were fixed before or after FcεRI stimulation. Scale bar = 5 µm. (TIF) [file pone.0111698.s002.tif]
